# Matrix remodeling associated 7 proteins promote cutaneous wound healing through vimentin in coordinating fibroblast functions

**DOI:** 10.1186/s41232-023-00256-8

**Published:** 2023-01-16

**Authors:** Ying Shen, Jinling Ning, Lu Zhao, Wei Liu, Ting Wang, Jie Yu, Yiqiang Wang

**Affiliations:** 1 National Clinical Research Center for Hematologic Disease, Jiangsu Institute of Hematology, The First Affiliated Hospital of Soochow University, Suzhou Medical College, Soochow University, Suzhou, 215006 China; 2 Department of Pathology, The First Affiliated Hospital of Soochow University, Soochow University, Suzhou, 215006 China; 3 Eye Institute of Xiamen University, Xiamen University, Xiamen, 361104 China; 4 Wisdom Lake Academy of Pharmacy, Xi’an Jiaotong-Liverpool University, Suzhou, 215123 China

**Keywords:** MXRA7, Vimentin, Fibroblast, ECM, Wound healing

## Abstract

Wound healing depends largely on the remodeling of the extracellular matrix around and reorganization of tissue-resident cells. Matrix remodeling associated 7 (MXRA7) is a member of the matrix remodeling-associated gene family and is involved in matrix remodeling-associated processes, such as inflammatory neovascularization, liver injury, and autoimmune skin disease. To investigate whether and how MXRA7 participate in cutaneous wound healing, an ear-punching model was utilized in wild-type (WT) and MXRA7-deficient mice, and the dermal fibroblasts from these mice were further studied in vitro. Results showed that the MXRA7 deficiency impaired the wound healing process in mice. Quantitative PCR indicated that lack of MXRA7 impaired the expression of several extracellular matrix genes (e.g., MMP-2) and inhibited signaling pathways (e.g., STAT3) in healing ear tissues. In in vitro culture system, migration, contraction, or proliferation of fibroblasts was impaired upon MXRA7 deficiency. Pull-down and mass spectrum assay revealed that vimentin was among the proteins that bound MXRA7 proteins in cells, and further investigations indicate MXRA7 was an autocrine factor in fibroblasts that involved vimentin in certain ways, such as JNK and STAT3/STAT5 signaling pathways in our study. In conclusion, MXRA7 proteins promote wound healing through vimentin in coordinating fibroblast functions.

**Supplementary Information:**

The online version contains supplementary material available at 10.1186/s41232-023-00256-8.

## Introduction

Tissue hemostasis is a dynamic and tightly regulated process that relies on coordinative interactions among tissue-resident cells and their environment, herein referred to as extracellular matrix (ECM). ECM is an organized structure formed by various components including those produced by the cells they embrace, and on the other side, ECM provides or delivers stimuli to cells required for their functions. Once a wound, either acute or chronic, biological or non-biological, the hemostasis is broken and diseases might follow. When a wound occurs in a functioning tissue, a healing process might be initiated. Effective healing leads to the regaining of tissue hemostasis functions. Surely enough, a better understanding of ECM and their producers promote a better understanding of the pathogenesis or management of diseases, and vice versa. Recently, we found that an ill-defined gene, matrix remodeling associated 7 (MXRA7), was involved in the pathogenesis of several disease models set up in the eyes [1], bones [2], livers [3], or skins [4], all via mechanisms apparently associated with matrix remodeling, just as its name implied. In those studies, we demonstrated that recombinant MXRA7 proteins modulated functions of cultured mesenchymal stem cells, prompting us to speculate that in physiological conditions, MXRA7 proteins might be secreted into extracellular matrix spaces, which would in turn modulate the resident cells. Should that hypothesis be true, for example in cutaneous tissue, MXRA7 might also play roles in other pathophysiological processes that occurred in skins besides the previously studied psoriasis [4].

Wound healing is a dynamic and strongly regulated process which associated with a sequence of events, including inflammation, migration, and proliferation of different cell types. Fibroblasts are critical in the wound repairing process, such as decomposing the fibrin clot and secreting new ECM to support other cells associated with effective wound healing [5]. Vimentin is a type III intermediate filament protein, and intermediate filaments induce changes in cell shape, adhesion, and migration [6]. Eckles et al. demonstrated vimentin is necessary for wound healing and found that the lack of vimentin impairs the motility of fibroblasts and wound healing in mice [7]. A study by Cheng et al. has demonstrated vimentin coordinated with fibroblast proliferation [8]. However, the specific mechanism remains to investigate.

The current study utilized ear punch as an acute wound model and demonstrated that MXRA7 was required for normal cutaneous wound healing and that MXRA7 proteins were truly secreted into extracellular spaces to participate in scaffold formation and overall cutaneous wound healing. Furthermore, we firstly demonstrated MXRA7 promoting wound healing through vimentin in coordinating fibroblast proliferation via p38/JNK and STAT3 signal pathway.

## Materials and methods

### Mice and cell lines

Heterozygous founders B6N-Mxra7^<tm1a(EUCOMM)Wtsi>/H^ on C57BL/6N background were obtained from the Medical Research Council (MRC, Swindon, UK). Male and female founders were cross-bred to generate the homozygous and wild-type breeders, which were genotyped according to the protocol provided by MRC (not detailed). All mice were maintained in a specific pathogen-free facility in Soochow University. Animal experimental protocols were approved by the Ethics Committee of Soochow University following the Guidelines on the Humane Treatment of Laboratory Animals (Ministry of Science and Technology of China, 2006). An SV40-transformed murine endothelial cell line, SVEC4-10 (China Center for Type Culture Collection, Wuhan, China), and the NIH3T3 mouse cell line (Shanghai Institute of Cell Biology, Shanghai, China) were cultured routinely and processed as described below.

### Ear punch wound healing model

A penetrating punch was made in the center of each ear of the mice by using a metal punch of 2 mm in diameter. Based on previous knowledge that both age and gender affect wound healing rate [9], only 6~8-week-old female mice were recruited into this study if not otherwise stated. At desired time points, the animals were euthanized and the ears were harvested. Pictures were taken and the areas of the punch holes were obtained with the software Image-Pro Plus 6.0 (Media Cybernetics, Rockville, MD).

### Histological analysis

Ears were harvested on day 7 and day 28 after punching and fixed in 4% paraformaldehyde for 24h followed by routine embedding. Continuous sectioning and hematoxylin and eosin staining were applied. To ensure the comparability among sections, an effort was made to pick the sections running across the hypothetical center of the original punch hole, and the amounts of infiltrates and new vessels were calculated properly. The sections were reviewed by a certified pathologist (WL).

### Immunohistochemical detection of MXRA7 proteins in murine tissues

At the time of experiments, tissues were harvested from mice and subjected to routine immunohistochemical assay. In brief, SZ181 (a monoclonal antibody against mouse MXRA7 produced in our lab) was used at a predetermined dilution (1:100) and incubated overnight at 4°C. HRP anti-rat/rabbit polymer (MXB, Fuzhou, China) was applied for 30 min at room temperature, and DAB reagent was used to envision the staining for 5–30 min. After washes with distilled water, the slides were counterstained with hematoxylin for 30 s and blued with PBS (pH 7.6). After drying and sealing, the images were captured.

### Quantification of genes at the mRNA level

A piece of tissue about 3 mm in diameter along the edge of injured tissues was collected and total RNA was extracted with RNAiso Plus (TaKaRa, Dalian, China). Total RNA was reverse transcribed inverted into first-strand cDNA using PrimeScript^TM^ II 1st Strand cDNA Synthesis Kit (TaKaRa, Dalian, China) according to manufacturer’s instructions. Real-time PCR (RT-PCR) assays with predetermined primers were performed on an ABI 7500 Fast RT-PCR System using TaqMan or SYBR-green protocols. Ct value for all genes and ΔCt=Ct_gene_−Ct_Actb_ for the target gene was obtained for each sample, and the relative level of a target gene in MXRA7^−/−^ group vs. WT group was calculated as 2^(ΔCt_WT_ − ΔCt_Mxra7_^−/−^). The primer sequences of target genes are listed in Table S1.

### Western blot assay of murine or human proteins

To compare the protein levels of different tissues or cells, the tissues or cells were harvested and lysed in RIPA buffer (Beyotime, Shanghai, China) with 1mM PMSF for 30 min on ice. The total protein samples were quantified and denatured by boiling in a loading buffer, and 30μg proteins of each sample were performed with SDS-PAGE. Then, the membranes were incubated with primary antibodies such as SZ181 (1:1000), polyclonal anti-human MXRA7 (1:1000 dilution; Sigma-Aldrich Company, St Louis, MO), and β-tubulin (1:1000 dilution, TransGen Biotech, Beijing, China) at 4°C overnight. Horseradish peroxidase-conjugated secondary antibodies (1:2000 dilutions, Cell Signal Technology, Beverly, MA) were added and incubated. The signals were detected using ECL and scanned in a Tanon 4600SF gel imaging system (Tanon Biotech, Shanghai, China). ImageJ software (NIH, Bethesda, MD) was used for the numerical quantification of blot results.

### Isolation and migration assay of murine skin fibroblasts

Primary mouse dermal fibroblasts were isolated from the skin of 2- to 3-day-old pups from WT and MXRA7^−/−^ mice. For migration assay, the cells were grown to confluence in a 24-well plate. A 100-μL tip was used to scratch across the cells and unattached cells were removed by PBS washing. Fresh DMEM without serum was added and a picture was taken for mark. After culturing for 24 h, another picture was taken at the same site. Image-Pro Plus was used to quantitate invaded areas.

### Cell adhesion assay

During the rapid growth phase, cells were resuspended at a density of 1.0 ×10^5^ cells/mL and added to each well precoated with collagen I and incubated at 37°C, 5% CO_2_. After seeding for 30min and 90min, unattached cells were washed twice with PBS. The cell adhesion was detected using CCK8 assay and FITC phalloidin staining (10 μg/mL, Sigma, St. Louis, MO).

### Proliferation assay

Fibroblasts at the exponential phase were harvested and reseeded into 96-well plates at3000 cells/well. At 24, 48, and 72 h, CCK8 reagents (bimake, Shanghai, China) were added (10 μL/well). One hour later, the plates were read for OD450. For EdU incorporation assay, 2×10^5^ cells were seeded in 24-well plates. Twenty-four hours later, the culture was switched to a medium without serum for starvation. Three hours later, the medium was replenished with a complete medium containing different concentrations of recombinant MXRA7 proteins (0, 5, 50, 500 nM) for 24 h. Then, 10 μM EdU solution was added to each well and co-cultured for another 2 h. Finally, labeling was performed with a Cell-Light TM 5′-ethynyl-2′-deoxyuridine Imaging Detection kit (RIBOBIO, Guangzhou, China) following the manufacturer’s protocol. Images were captured using an Olympus IX51 inverted fluorescence microscope and analyzed using Image-Pro Plus 6.0.

### Pull-down and mass spectrum of MXRA7-binding proteins

To search for the potential receptor(s) or interactor(s) of MXRA7, a pull-down experiment was performed. Total proteins prepared from SVEC4-10 cells or NIH3T3 cells were divided into three equal parts, one of which was left as input control, and another two parts were subjected to pull-down as above. Then, the three samples were resolved on SDS-PAGE and we use mass spectrum to identify the composition of proteins isolated from a gel region. Subsequently, to confirm the actual presence of hypothetical potential receptor of MXRA7 (e.g., vimentin), the samples were subjected to blotting with anti-vimentin antibody (1:2000 dilution) or anti-GST antibody (1:1000 dilution; TransGen Biotech, Beijing, China) as primary antibodies.

### Co-immunoprecipitation

NIH3T3 cells were harvested into Co-IP buffer (Tris-HCl 50mM, pH7.4, MgCl_2_ 10mM, NaCl 150mM, TritonX-100, 0.5%) supplemented with protease inhibitors and centrifuged at 12,000 g for 15 min. The supernatant was mixed with protein A/G agarose (Beyotime Biotechnology, Shanghai, China) at 4°C for 30 min to remove nonspecific binding constituents. Eight hundred micrograms of recovered proteins was mixed with 1μg anti-MXRA7 antibody (SZ181) or anti-vimentin antibody or isotype control IgG (Beyotime, Shanghai, China) at 4°C overnight on a roller. Then, 40μL protein A/G agarose beads were added for another 2 h at 4°C. The beads were spin-washed with pre-cooled Co-IP buffer six times. The final sediments were resuspended in an SDS loading buffer and boiled for 10 min. Cleared samples were separated on SDS-PAGE followed by transferring to the NC membrane. Routine WB was performed with an anti-MXRA7 antibody (1:1000 dilution, Sigma-Aldrich Company, St Louis, MO) or anti-vimentin antibody (1:1000 dilution, Proteintech, Wuhan, China).

### Immunofluorescence staining

For immunofluorescence staining, the NIH3T3 cells were cultured in 24-well plates on coverslips; when cells entered the exponential growth phase, they were fixed with 4% PFA. All the samples were permeabilized with 0.5% Triton. To observe whether MXRA7 proteins colocalize with vimentin, anti-murine MXRA7 (1:100 dilution, SZ181) and anti-vimentin (1:500 dilution, Abcam, Cambridge, MA) antibodies were used and incubated overnight at 4°C. Alexa Fluor 488-conjugated anti-rabbit IgG (1:500, Abcam, Cambridge, MA) and Alexa Fluor 594 anti-mouse IgG (1:500, Abcam, Cambridge, MA) were used as the secondary antibody and incubated for 1 h at room temperature. The images were acquired with a microscope (Leica, Bensheim, Germany). 

### Statistical analysis

When applicable, the data are presented as the means ± S.D. The statistical analyses were performed with Student’s *t* test (unpaired, two-tailed). *p*-values <0.05 were considered significant.

## Results

### Regenerating cutaneous tissues manifested increased MXRA7 expression

**Fig. 1:**
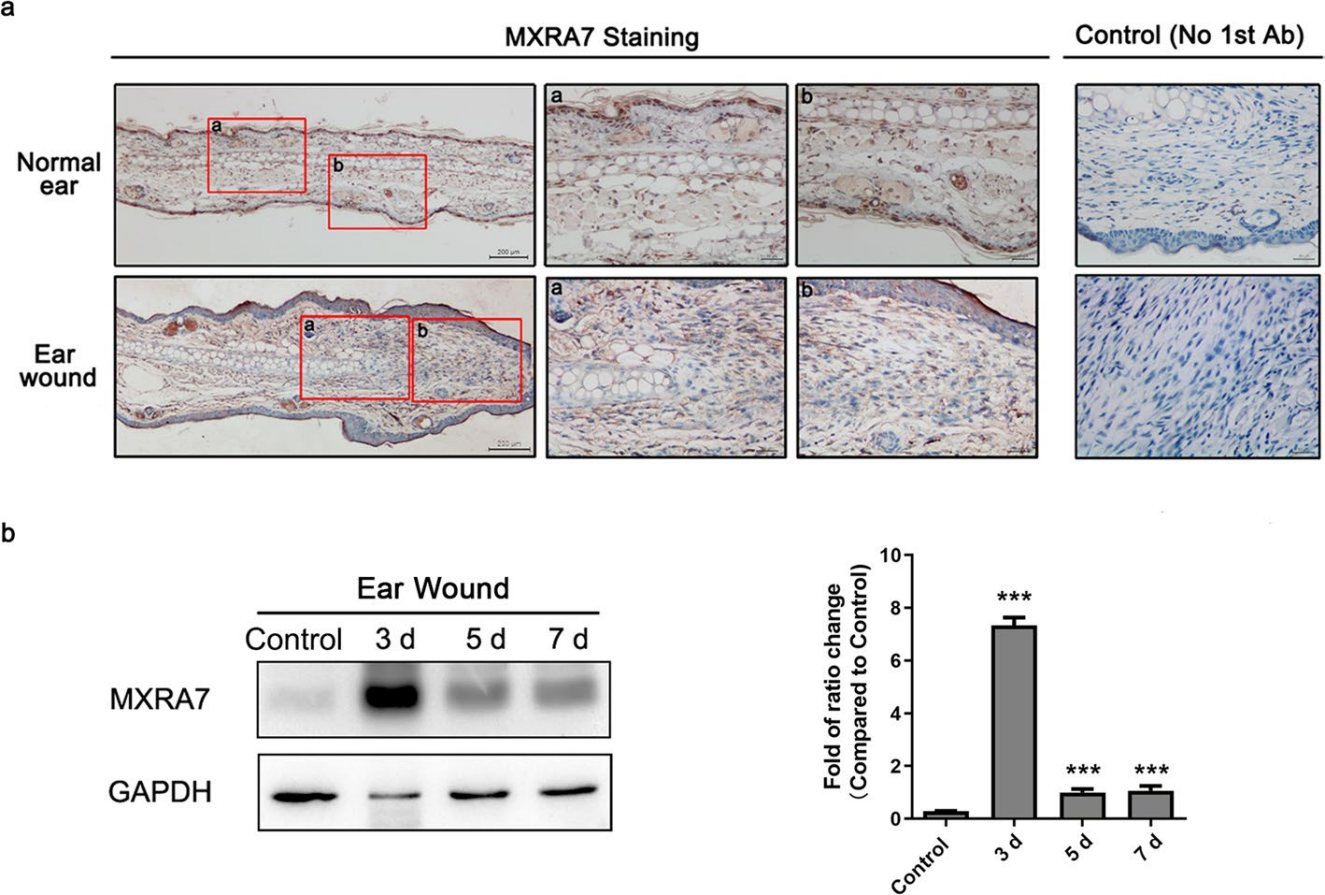
The expression and distribution of MXRA7 at different stages of wound healing. **a** Detection of MXRA7 proteins in the normal ear or in tissues close to an ear punch. **b** MXRA7 protein levels in tissues around the punch at different times as detected by western blot and relative quantification statistics. *p<0.05, **p<0.01, ***p<0.001

The ear punch hole made with a puncher is an excellent model for studying cutaneous wounds and healing for its convenience and consistency and thus was selected to explore the potential effects of MXRA7 in wound healing. In wild-type (WT) mice, when a punch hole was made, the dermal layers of the regenerating section manifested decreased expression of MXRA7 overall, and MXRA7 proteins were more likely distributed in non-cellular structure, assumedly intercellular spaces. In the cutaneous mass of regenerating tissues, the fibroblast cells stained heavily for MXRA7 (Fig. 1a). At the same time, western blot assay of proteins from injured area tissues showed that the MXRA7 expression increased significantly after the wound, especially at day 3 post-injury (Fig. 1b). These observations suggested that MXRA7 proteins might be an initiator or enhancer of the wound healing process.

### Deficiency of MXRA7 impaired wound healing process in mice

**Fig. 2:**
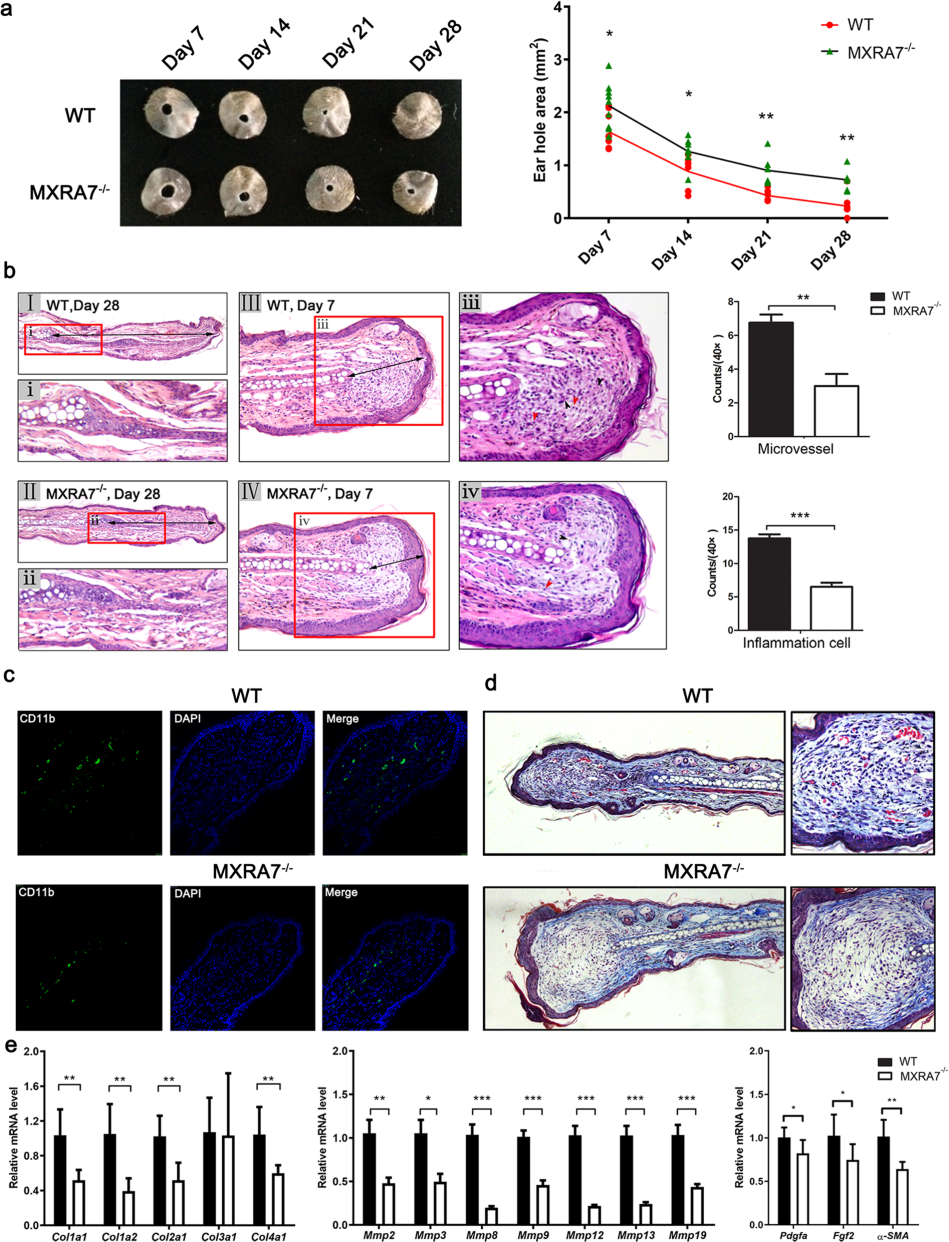
Wound healing was hampered in MXRA7^−/−^ mice. **a** Comparison of wound healing in WT and MXRA7^−/−^ mice as indicated by residual punch sizes. Shown were representative images of ears and sizes of punch holes calculated with the Image-Pro Plus 6.0 (average with a standard deviation of seven samples from seven mice in each group). **b** Representative histological images of healing ear tissues. (III, IV) At day 7 after injury, the newly regenerating tissue contained more infiltrating cells (red arrow heads) and new blood vessels (black arrow heads) in WT mice than in MXRA7^−/−^ mice. (I, II) Double-headed arrows measured the length of regenerated tissues by day 28. Original magnifications: I, II, 100×; I II, IV, i, ii, 200×; iii, iv, 400×. *p<0.05, **p<0.01, ***p<0.001. **c** Direct immunofluorescence staining of CD11b showed that inflammatory cells of WT were significantly more than those of MXRA7^−/−^ mice on day 7 after injury. **d** Masson staining showed there was less collagen deposition in the regenerated tissues of MXRA7^−/−^ mice compared to WT mice on day 19 after injury. **e** RT-qPCR detected the expression of genes coding for collagens, MMPs, and myofibroblast differentiation modulators

In line with the above notations, wound healing in MXRA7^−/−^ mice was slower than that in WT counterparts. Specifically, on day 28 after injury, the punch holes in most WT mice were no longer visible to the naked eyes, while apparent holes were still present in MXRA7^−/−^ mice (Fig. 2a). Though there were controversial reports on the effect of sex on healing speed [10], ear punch wounds in female mice of both MXRA7^−/−^ and WT mice healed slightly faster than those in male ones, but no statistical significance was achieved in either sex (Fig. S1).

Consistent with the observation of gross examination, newly regenerated tissues at a histological level in MXRA7^−/−^ mice were shorter than that in WT mice at day 7 and day 28 after injury, respectively (Fig. 2b). Furthermore, regenerated tissues in MXRA7^−/−^ mice manifested fewer newly formed vessels and fewer infiltrated inflammatory cells, indicating a more active regenerating presentation in WT mice (Fig. 2b, c). Masson staining showed the collagen deposition in MXRA7^−/−^ mice was decreased at day 19 after injury (Fig. 2d). To identify the molecular basis of the impairment of wound healing in MXRA7^−/−^ mice, three groups of genes were measured at mRNA level in the reconstituting ear tissues on day 7 after injury. These genes coded for collagens, matrix metalloproteinase (MMPs), and myofibroblast differentiation modulators. It was found that with exception of *col3a1*, the expression of all other genes manifested a decrease in injured MXRA7^−/−^ mouse ears when compared with WT ones (Fig. 2e). All of these results indicated MXRA7 may influence wound healing by regulating the function of fibroblasts.

### Deficiency of MXRA7 impaired proliferation and migration but enhanced adhesion and contraction of fibroblasts

**Fig. 3:**
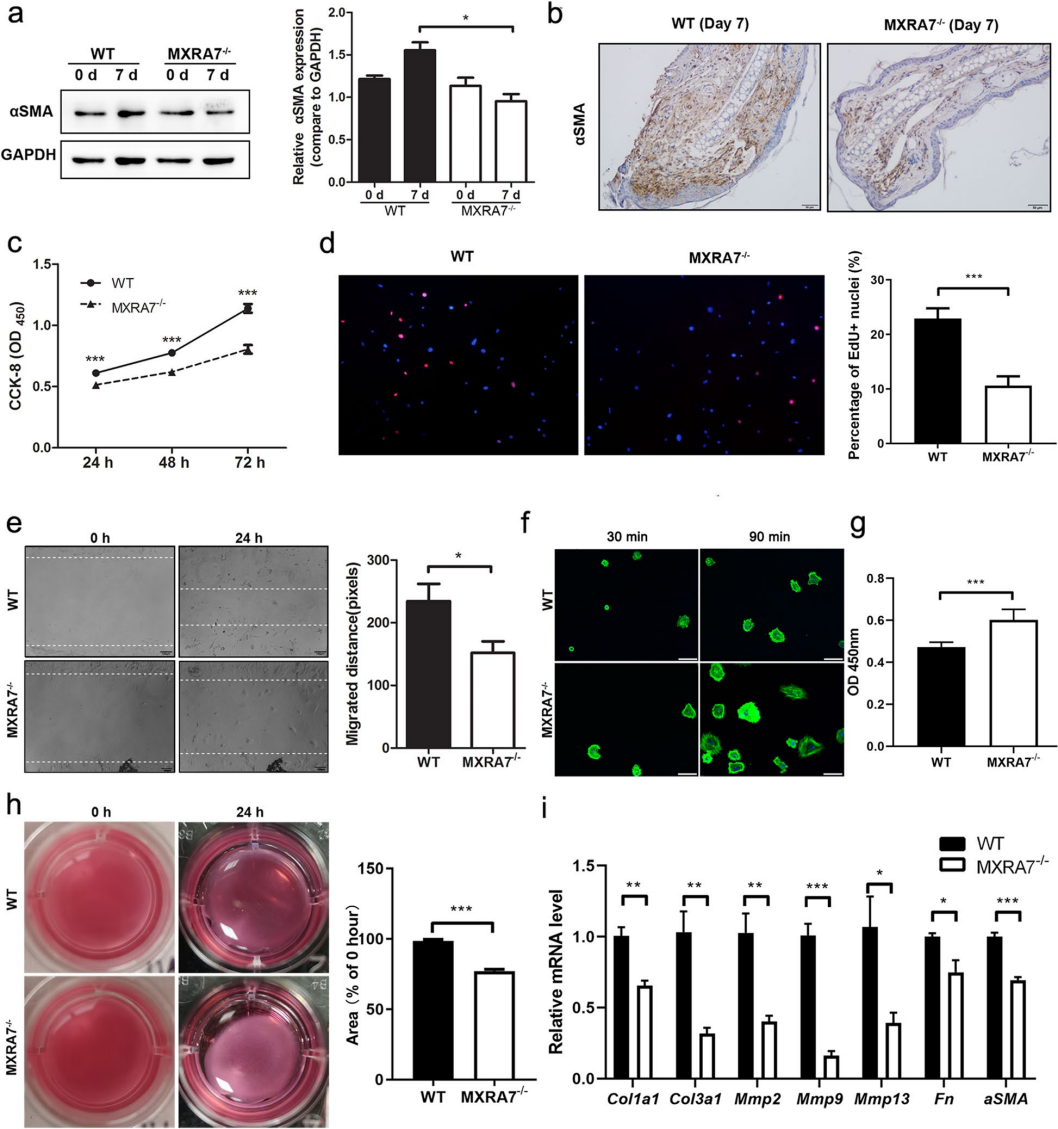
Functions of fibroblasts were impaired with MXRA7 deficiency. **a**, **b** Western blot and IHC staining for αSMA in tissue belts around the punch holes. **c**, **d** Fibroblasts purified from abdominal skins of WT and MXRA7^−/−^ mice were compared for their proliferation as measured with CCK-8 assay and EdU incorporations. **e** Fibroblasts purified from the abdominal skins of WT and MXRA7^−/−^ mice were compared for their migration ability. **f** Confocal microscopy images of WT and MXRA7^−/−^ fibroblast cells. Cells were cultured in a collagen-precoated 24-well plate for 3 and 90 min, and F-actin was stained with FITC-labeled phalloidin. **g** CCK8 assay was used to detect the number of cells adhered to collagen. **h** Contraction behavior of cell-seeded collagen gels for WT and MXRA7^−/−^ fibroblasts. WT and MXRA7^−/−^ fibroblasts were embedded in collagen gel and incubated for 24 h. After photography, collagen gel contraction was evaluated by measurement of gel diameter. **i** RT-qPCR detected the expression of genes coding for collagens, MMPs, fibronectin, and αSMA in WT and MXRA7^−/−^ fibroblasts. Error bars represent the mean ± S.D.; *p<0.05, **p<0.01, ***p<0.001

Quite a few public datasets had shown that fibroblasts were among the cells that express MXRA7 most abundantly in the skin (Fig. S2, [11]). Meanwhile, combined with the distribution changes of MXRA7 observed during wound healing in WT mice (Fig. 1a), we speculated MXRA7 may play important roles on dermal fibroblasts during wound healing. To check the role of MXRA7 in fibroblasts in cutaneous wound healing, a couple of features related with fibroblasts were examined. First, the expression of the fibroblast-specific activation marker αSMA was measured. Western blotting showed that αSMA was increased in healing tissues of WT ears at day 7, and this increase was abrogated in MXRA7^−/−^ mice (Fig. 3a). The decrease of αSMA expression in MXRA7^−/−^ mice compared with WT mice was confirmed with immunohistochemistry (Fig. 3b). As expected, αSMA manifested a higher expression level in the growing edge of the regenerating ear tissues in WT mice, but its intensity was decreased in MXRA7^−/−^ mice. Next, dermal fibroblast cells were isolated from mice for in vitro comparison, and it was found that fibroblasts from MXRA7^−/−^ mice showed decreased proliferation potential (Fig. 3c, d). Migration of fibroblasts was also impaired upon MXRA7 deficiency (Fig. 3e). Cell spreading assay was performed to compare the adhesion of MXRA7^−/−^ and WT fibroblast to collagens, and the results showed that MXRA7 deficiency enhanced fibroblast adhesion (Fig. 3f, g). A similar increase in contractility was observed for MXRA7^−/−^ fibroblasts (Fig. 3h). RT-qPCR assay showed that besides fibronectin and αSMA, the expression of common ECM-related collagens and matrix metalloproteinase (MMPs) were decreased in MXRA7^−/−^ fibroblasts. All these suggested that MXRA7 is required for fibroblast proliferation and migration and plays roles in regulating ECM (Fig. 3i).

### Vimentin-mediated MXRA7 functions in fibroblasts

**Fig. 4:**
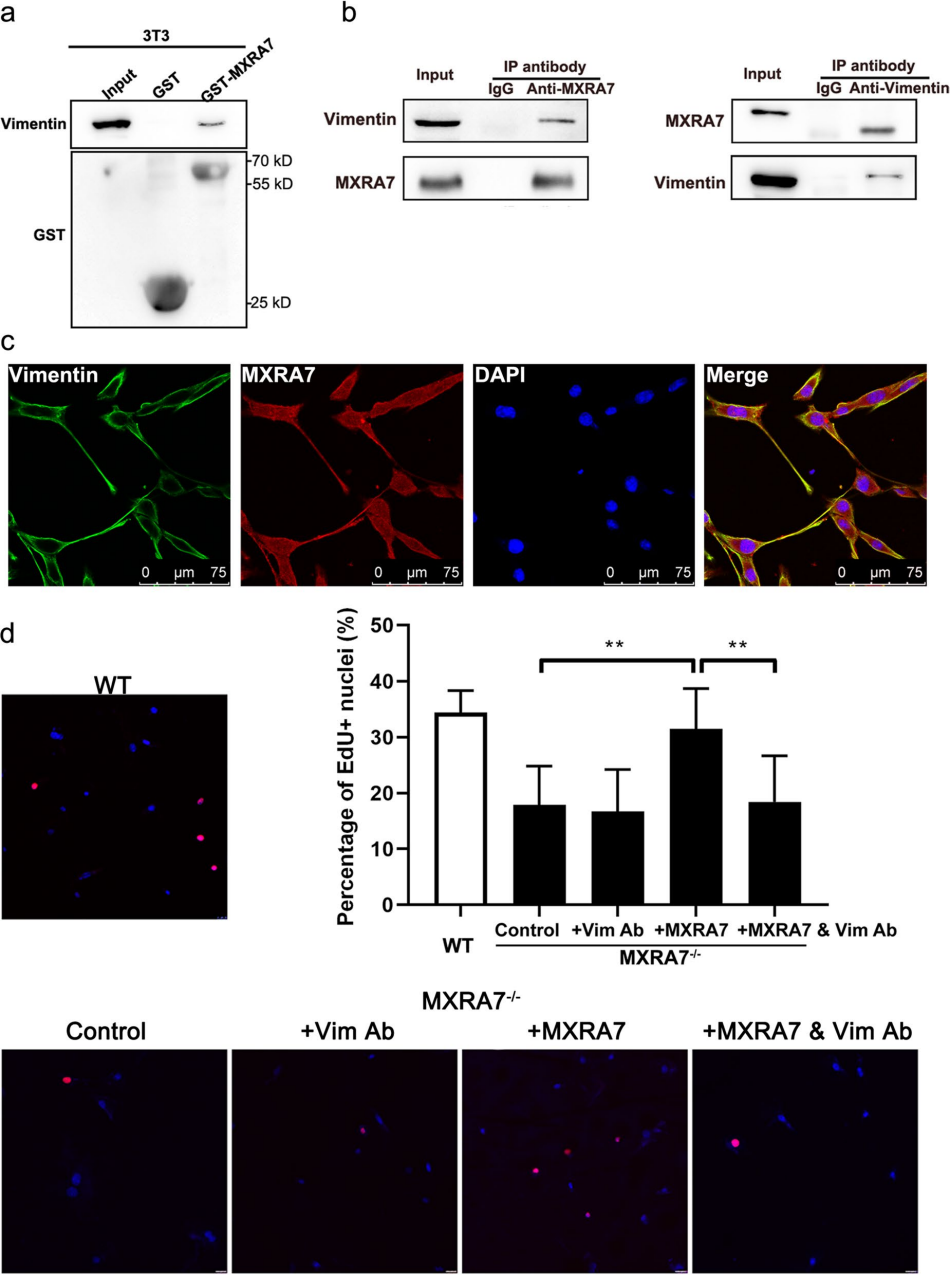
MXRA7 proteins manifested direct interactions with Vimentin. **a** Membrane proteins from NIH3T3 fibroblasts were subjected to pull-down, and recovered samples were separated on SDS-PAGE followed by staining for vimentin or GST control. **b** Total proteins prepared from NIH3T3 cells were precipitated with anti-MXRA7 antibodies or anti-vimentin, respectively, and sediments were cross-examined by western blot. **c** The co-localization of vimentin and MXRA7 proteins in NIH3T3 cells was detected by a confocal laser microscope. **d** Vimentin antibody can block the proliferative effect on MXRA7^−/−^ fibroblasts caused by the protein rmMXRA7

In a parallel project performed in vascular endothelial cells, we obtained data showing that vimentin (Vim) was among the proteins that formed a complex with MXRA7 proteins in murine endothelial cells (Table S1, Fig. S3). This MXRA7-Vim interaction was also observed in the murine fibroblast cell line NIH3T3. In brief, rmMXRA7 could pull down Vim from proteins of NIH3T3 (Fig. 4a). When NIH3T3 cellular proteins were immune precipitated with anti-MXRA7 antibodies, vimentin was also detectable in the precipitate as expected, or vice versa (Fig. 4b), confirming that Vim and MXRA7 formed a complex in physiological conditions. Immunostaining demonstrated that while Vim was mainly distributed in sub-plasma membrane spaces, MXRA7 were distributed evenly in cytosolic with increased levels on the membrane (Fig. 4c). Meanwhile, the rescuing effect of rmMXRA7 on fibroblast proliferation in a concentration-dependent manner (Fig. S4) was blocked by anti-Vim antibodies (Fig. 4d).

**Fig. 5:**
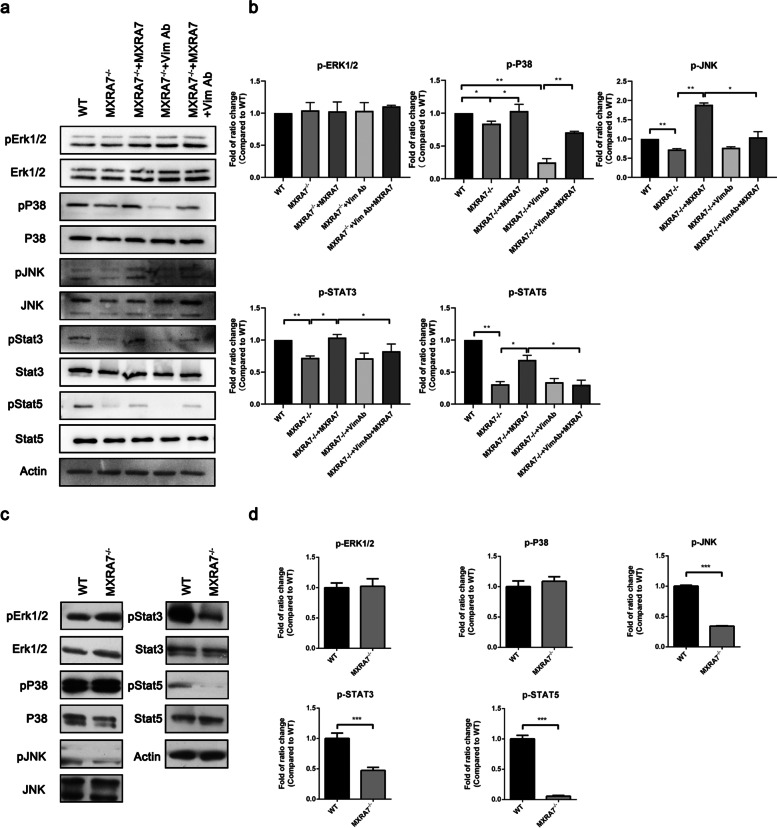
Western blot detected the signaling pathways that participate in wound healing. **a** Including the phosphorylation level of the MAPK pathway (ERK1/2, JNK, and p38) and STAT3/STAT5 pathways from isolated fibroblasts. **b** Summary of western blotting results in **a**. Error bars represent the mean ± S.D.; *p<0.05, **p<0.01, ***p<0.001. **c** The phosphorylation level of MAPK pathway (ERK1/2, JNK, and p38) and STAT3/STAT5 pathways from wounds at day 7 after injury. **d** The bands in **c** were quantified by using ImageJ. Error bars represent the mean ± S.D.; *p<0.05, **p<0.01, ***p<0.001

Next, we measured whether the MXRA7-Vim interaction affected the signaling pathways reported to participate in wound healing. It was found that JNK, P38, and STAT3/STAT5 pathways were impaired in MXRA7^−/−^ fibroblast while the ERK1/2 pathway was not affected in this context. After the addition of exogenous rmMXRA7 proteins, the levels of pP38, pJNK, pSTAT3, and pSTAT5 were up-regulated in MXRA7^−/−^ fibroblasts (Fig. 5a, b). Not surprisingly, the addition of anti-Vim antibodies blocked the rescuing effect of rmMXRA7 for pJNK, pSTAT3, and pSTAT5 pathway activation. The “hypersensitivity” of pP38 to anti-Vim antibodies was beyond our knowledge and explanation. At the same time, we also found that JNK, STAT3, and STAT5 pathways were impaired at day 7 after injury in MXRA7^−/−^ mice (Fig. 5c, d). Therefore, we believe that the changes of these three phosphorylated proteins at the wound site may be due to their effect on fibroblasts. These results suggested that the MXRA7 deficiency impaired endogenous or baseline activation of JNK and STAT3/STAT5 signaling pathways, which might underline the alteration observed in MXRA7-deficient mice or cells.

## Discussions

Successful cutaneous wound healing requires a series of tightly coordinated steps including inflammation, ECM remodeling, and tissue reconstruction. In all these processes, fibroblasts are among the main effector cells [12]. Many public datasets obtained via single-cell sequencing demonstrated that fibroblasts are among the cells that contained the most MXRA7 messengers in examined tissues (Fig. S2). The facts that soluble rmMXRA7 proteins rescued functions of MXRA7^−/−^ fibroblasts and that anti-Vim antibodies blocked the rmMXRA7’s rescuing potency (Figs. 4 and 5) suggested that MXRA7 was an autocrine factor in fibroblasts that involved Vim in certain ways. Though lacking deep investigation into the molecular mechanisms underlying MXRA7 functions, evidence was strong enough to suggest that MXRA7 was a novel player in cutaneous wound healing and thus deserved more investigations in the future, and a couple of issues warrant discussions.

MXRA7 were multifaceted molecules and may function in various manners. MXRA7 could be secreted out of cells and, in turn, concur effect on cells, just like in fibroblasts in this study. In cells, MXRA7 may interact with Vim, thus regulating cell moving or cytoskeleton reorganization. Obviously, MXRA7 proteins did not stay in cells merely as loads produced in ER/Golgi waiting to be secreted out. The ability of MXRA7 proteins to bind Vim and probably other cytoskeleton molecules like TUBB5, ACTG1, etc., or metabolic enzymes like GAPDH, ATP5A1, etc. (Table S1), strongly suggested that MXRA7 should be an intracellular structural component as well. As one of the main intermediate filaments, Vim not only plays critical and meticulous roles in the cytoskeleton system [13], but also modulates communications between extracellular and intracellular scaffolds [14, 15], hence determining the overall cellular functions and body health [16, 17]. So, the discovery of direct interactions between MXRA7 and Vim provided an ideal starting point for dissecting the roles of cytosolic MXRA7 proteins.

Cutaneous healing is a procedure contributed by fibroblasts and keratinocytes. When cartilage was also involved, like in the ear punch model in this study, chondrocytes might also be modulated by MXRA7 just like fibroblasts. Comparing with the questions answered by this study, much more questions remained to be answered for a better understanding of how MXRA7 affected cutaneous healing at different levels. Future studies should define how MXRA7 proteins interact with well-known ECM constituents, how MXRA7 affects keratinocytes, and how MXRA7 participates in the regulation of various physiological and pathological processes concerning skin and beyond. More importantly, much more effort should be made to check whether the observations obtained in the murine system would be true for humans. Better knowledge of MXRA7 biology might provide new therapeutic insights in wound healing. More biomedical researches should address MXRA7’s potential involvement in the pathogenesis of matrix-related diseases or the potential utilization of MXRA7 as a target for such diseases*.*

From the general biological viewpoint, the significance of current findings went beyond cutaneous wound healing and also proposed MXRA7 as a valuable target and tool in the field of matrix biology. In one word, this study for the first time identified MXRA7 protein as a novel matrix constituent that might have other functions as well.

## Supplementary Information

**Additional file 1:** **Table S1.** Primers and probes used for RT-qPCR. **Table S2.** List of proteins pulled-down by recombinant MXRA7 from SVEC4-10 cells. **Figure S1.** The effect of genders on wound healing speed in both WT and MXRA7^-/-^ mice. **Figure S2.** Archived MXRA7 expression in various cells of different organs in Human Protein Atlas. **Figure S3.** GST pull-down analysis of the interaction between MXRA7 and Vimentin proteins by SDS-PAGE. **Figure S4.** EdU proliferation assay analysis of the effect of different concentrations of rmMXRA7 on the growth of MXRA7^-/-^ fibroblast cells.

## Data Availability

Data sharing is not applicable to this article. All data supporting the findings are presented within the article and in its Supplementary Materials.
