# Supplementary material for: Matrix remodeling associated 7 proteins promote cutaneous wound healing through vimentin in coordinating fibroblast functions
Source: Inflamm Regen. 2023 Jan 16;43:5. doi: 10.1186/s41232-023-00256-8 (PMC9841631; doi:10.1186/s41232-023-00256-8)
Supplement: Supplementary file 1 — Additional file 1: Table S1. Primers and probes used for RT-qPCR. Table S2. List of proteins pulled-down by recombinant MXRA7 from SVEC4-10 cells. Figure S1. The effect of genders on wound healing speed in both WT and MXRA7-/- mice. Figure S2. Archived MXRA7 expression in various cells of different organs in Human Protein Atlas. Figure S3. GST pull-down analysis of the interaction between MXRA7 and Vimentin proteins by SDS-PAGE. Figure S4. EdU proliferation assay analysis of the effect of different concentrations of rmMXRA7 on the growth of MXRA7-/- fibroblast cells. [file 41232_2023_256_MOESM1_ESM.docx]

**SUPPLEMTENTARY MATERIALS**

**Matrix remodeling associated 7 proteins promote cutaneous wound healing through vimentin in coordinating fibroblasts functions**

***Correspondence author at:** Wisdom Lake Academy of Pharmacy, Xi'an Jiaotong-Liverpool University, Suzhou 215123, China. Email address: yiqiang.wang@xjtlu.edu.cn.

| **Gene symbol** | **Description** | **-10lgP*** | **Coverage (%)** | **#Peptides** | **#Unique** |
| --- | --- | --- | --- | --- | --- |
| Tubb5 | tubulin, beta 5 class I | 309.93 | 76 | 70 | 12 |
| Gapdh | glyceraldehyde-3-phosphate dehydrogenase | 264.3 | 59 | 20 | 20 |
| Atp5a1 | ATP synthase, H+ transporting, mitochondrial F1 complex, alpha subunit 1 | 261.5 | 48 | 33 | 33 |
| Slc25a4 | solute carrier family 25 (mitochondrial carrier, adenine nucleotide translocator), member 4 | 252.61 | 62 | 40 | 14 |
| Vim | vimentin | 222.34 | 54 | 26 | 24 |
| Eef1a1 | eukaryotic translation elongation factor 1 alpha 1 | 219.08 | 45 | 18 | 18 |
| Actg1 | actin, gamma, cytoplasmic 1 | 205.92 | 54 | 14 | 6 |
| Samm50 | SAMM50 sorting and assembly machinery component | 203.95 | 46 | 18 | 18 |

**Table S2 List of proteins pulled-down by recombinant MXRA7 from SVEC4-10 cells ^#^.**

Note

# Methods:

* Only those genes with a -10lgP over 200 were listed here. Genes with very similar names or structures were omitted, such as tubulin alpha(s), other tubulin beta(s), Atp5b, or Slc25a3 etc.

**Table S1 Primers and probes used for RT-qPCR**

| **Gene symbol (accession number)** | **Primer and probe sequence (5′-3′)** | **Amplicon size** |
| --- | --- | --- |
| *Mmp-2* | F- CTGGGAGCATGGAGATGGATA | 96bp |
| (NM_008610) | R- AAGTGAGAATCTCCCCCAACAC |  |
|  | P- ACATGCCTTTGCCCCGGGCA |  |
| *Mmp-3* | F- CTATTCCTGGTTGCTGCTCA | 120bp |
| (NM_010809） | R- GAGATGGAAACGGGACAAGT |  |
|  | P- TGAACTTGGCCACTCCCTGGG |  |
| *Mmp-8* | F- CAATTCCGGTCTTCGAGGAA | 85bp |
| (NM_008611) | R- TCCCAGTCTCTGCTAAGCTGAA |  |
|  | P- CCACGATGGTTGCAGAGAAGCTTAAAGA |  |
| *Mmp-9* | F- GGGTCTAGGCCCAGAGGTAA | 86bp |
| (NM_013599) | R- AGACACGCCCCTTGCTGA |  |
|  | P- CCACGTCAGCGGGCTTCTCCC |  |
| *Mmp-12* | F- GAAGCAACTGGGCAACTGG | 106bp |
| (NM_008605) | R- ATCTTGACCTCTGGGGCACT |  |
|  | P- CAACTCAACTCTGGCAATAATGCACATCC |  |
| *Mmp-13* | F- AAGTGTGACCCAGCCCTATC | 156bp |
| (NM_008607) | R- CACATGGTTGGGAAGTTCTG |  |
|  | P- CTTCTGGCGCCTGCACCCTC |  |
| *Mmp-19* | F- AGGATACTGGCAATGGGATGA | 70bp |
| (NM_0011641) | R- GAGGGTCGGTCTGGCACTC |  |
|  | P- CTGGCCAGAACTGACCTTAGCCGCTA |  |
| *Col1a1* | F- CTGACTGGAAGAGCGGAGAGTAC | 260bp |
| ([NM_007742](https://www.ncbi.nlm.nih.gov/entrez/viewer.fcgi?db=nucleotide&id=927028864)) | R- GGTCAGCTGGATAGCGACATC |  |
|  | P- AGAACTGGTACATCAGCCCGAACCCC |  |
| *Col1a2* | F- TGGTGGCAGCCAGTTTGA | 145bp |
| ([NM_007743](https://www.ncbi.nlm.nih.gov/entrez/viewer.fcgi?db=nucleotide&id=958157208)) | R- CTCATCCAGGTACGCAATGC |  |
|  | P- TGCTTCTCAGAACATCACCTACCACTGCA |  |
| *Col2a1* | F- GGTGGCTTCCACTTCAGCTAT | 105bp |
| ([NM_001113515](https://www.ncbi.nlm.nih.gov/entrez/viewer.fcgi?db=nucleotide&id=169658374)) | R- GATGTTCTGGGAGCCCTCAGT |  |
|  | P- ACCTGGCTCCCAACACCGCTAACGT |  |
| *Col3a1* | F- GGCAGTGATGGGCAACCT | 86bp |
| ([NM_009930](https://www.ncbi.nlm.nih.gov/entrez/viewer.fcgi?db=nucleotide&id=226423932)) | R- GGTCCAACTTCACCCTTAGCA |  |
|  | P- CCCCCTGGCCCTCCTGGAACT |  |
| *Col4a1* | F- ATTAGCAGGTGTGCGGTTTG | 77bp |
| ([NM_009931](https://www.ncbi.nlm.nih.gov/entrez/viewer.fcgi?db=nucleotide&id=161484653)) | R- CACTGCGGAATCTGAATGGT |  |
|  | P- AGCACCGGCCATGGTGATGGC |  |
| *α-SMA* | F- GGTGACGAAGCACAGAGCAA | 74bp |
| ([NM_001272041](https://www.ncbi.nlm.nih.gov/entrez/viewer.fcgi?db=nucleotide&id=439253892)) | R- CAGTTGGTGATGATGCCATGTT |  |
|  | P- AGAGGAATCCTGACCCTGAAGTACCC |  |
| *Actb*  (NM_007393) | F-GCAAGCAGGAGTACGATGAG  R-CCATGCCAATGTTGTCTCTT | 148bp |
|  | P-TCCATCGTGCACCGCAAGTG |  |
| *Pdgfa* | F- TGTAACACCAGCAGCGTCAAGT | 159bp |
| ([NM_008808](https://www.ncbi.nlm.nih.gov/entrez/viewer.fcgi?db=nucleotide&id=118130644)) | R- GTTCAGGTTGGAGGTCGCACAT |  |
| *Fn1* | F- ACCAGGTTGATGATACTTCC | 198bp |
| (NM_010233) | R- TCTCCTCCACAGCATAGATAG |  |
| *Fgf2* | F- GGCTGCTGGCTTCTAAGTGT | 112bp |
| ([NM_008006](https://www.ncbi.nlm.nih.gov/entrez/viewer.fcgi?db=nucleotide&id=159032535)) | R- AGTGCCACATACCAACTGGAG |  |
| *Actb*  (NM_-_007393) | F:GCTCCTAGCACCATGAAGAT  R:GTGTAAAACGCAGCTCAGTA | 197bp |

**
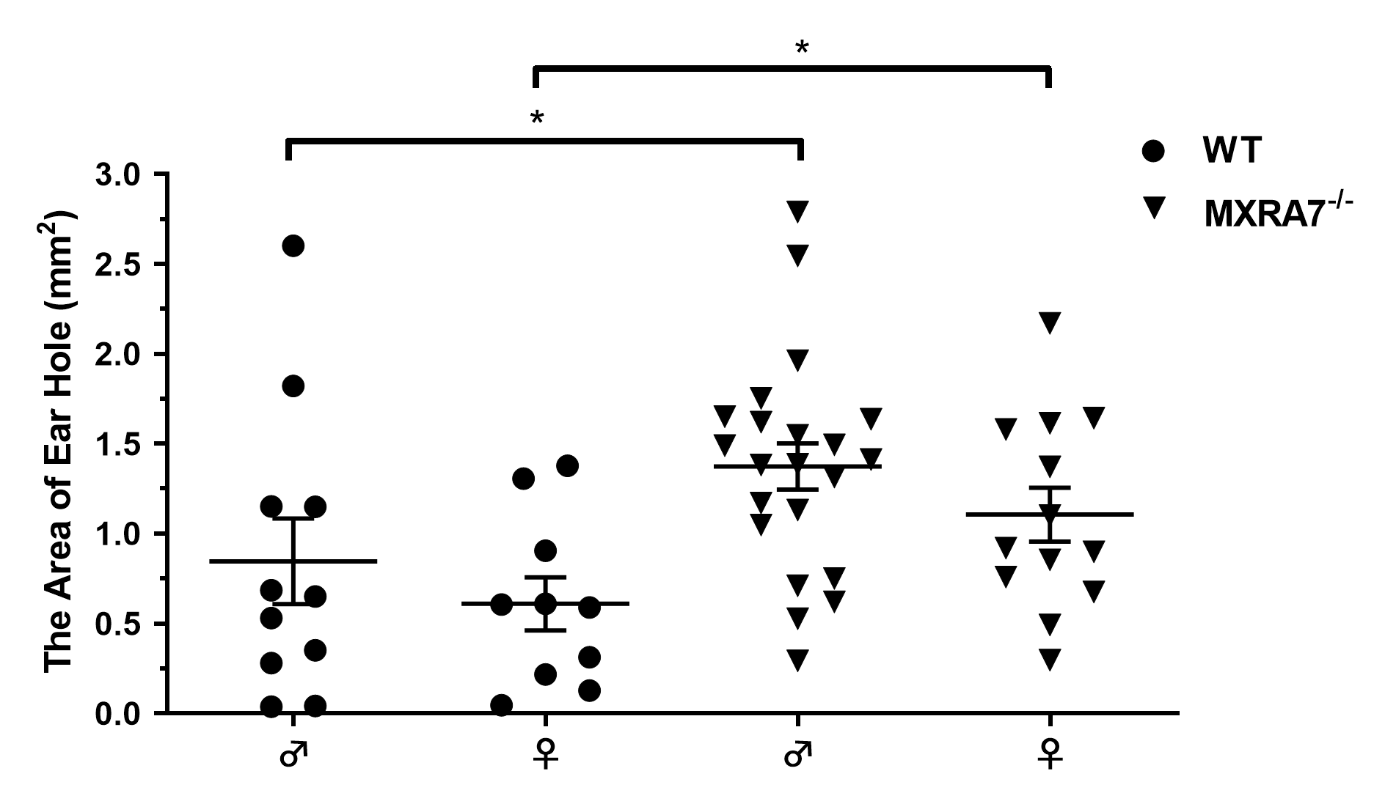
**

**Figure S1. The effect of genders on wound healing speed in both WT and MXRA7^-/-^ mice.** Shown was one representative of two experiments with similar results. Each mark represented an ear and the lines the average with standard deviations of all samples in that group.


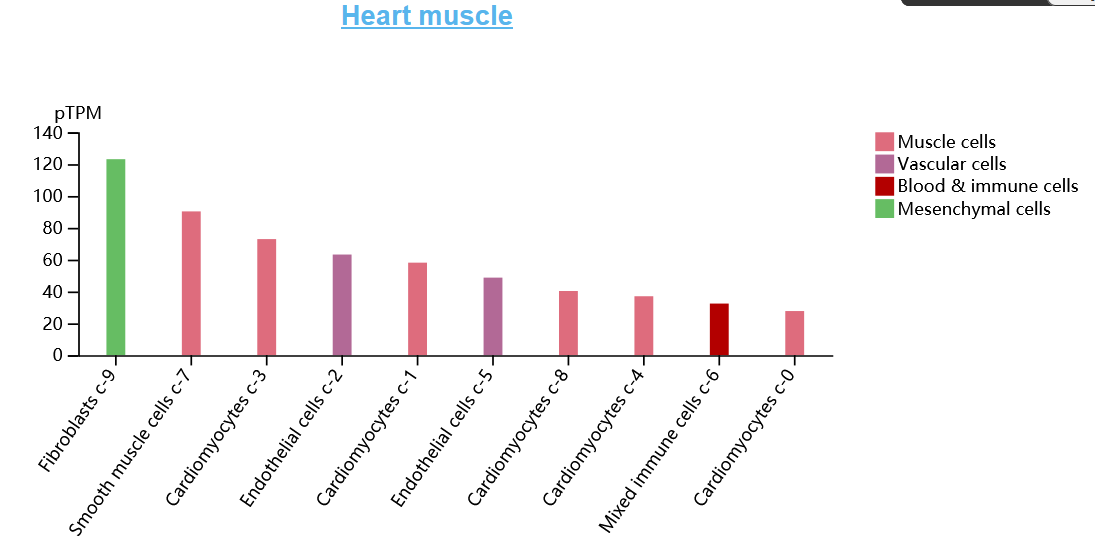

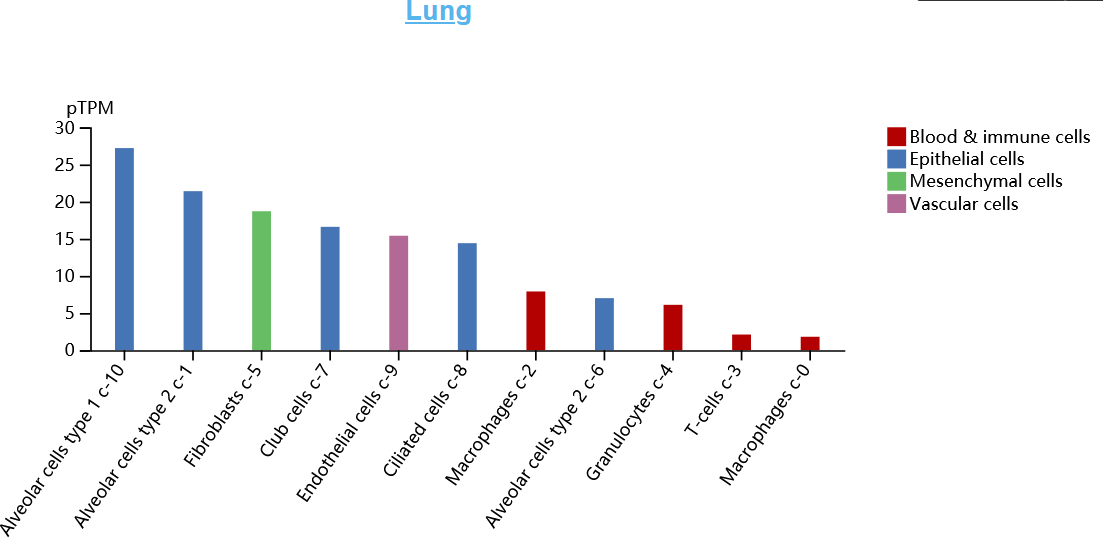


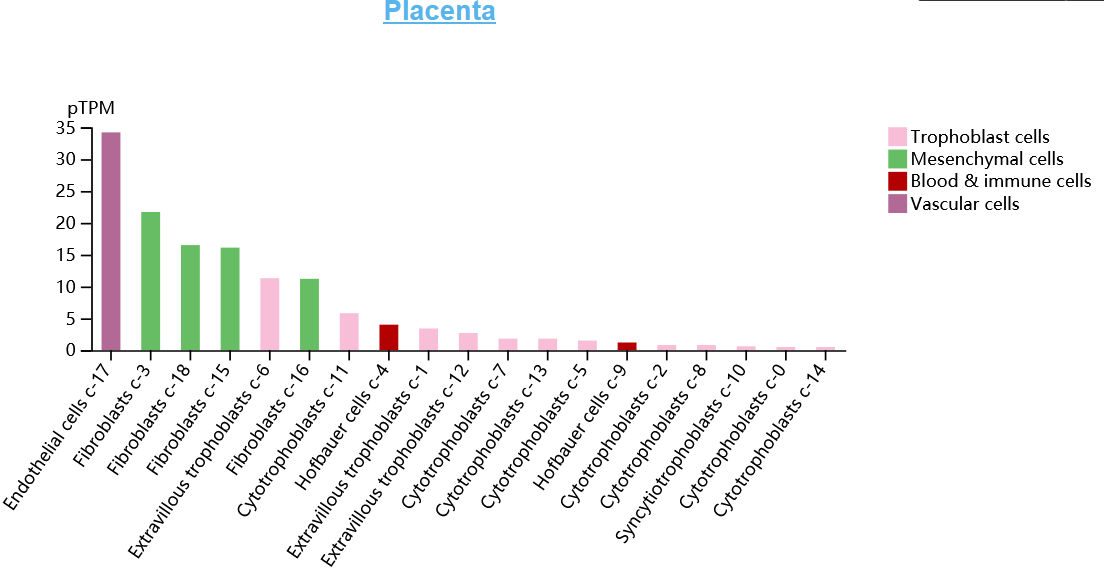

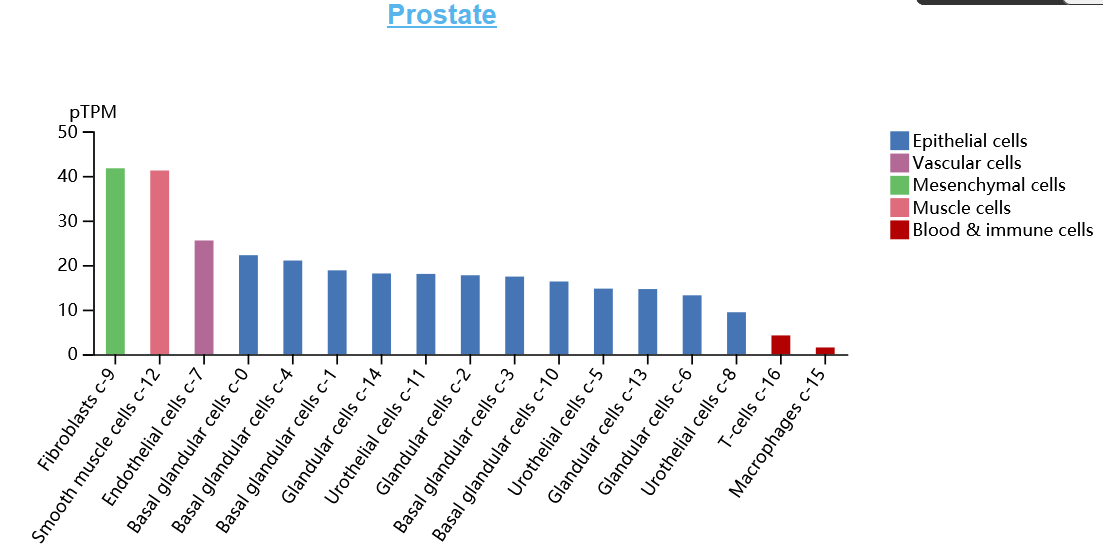


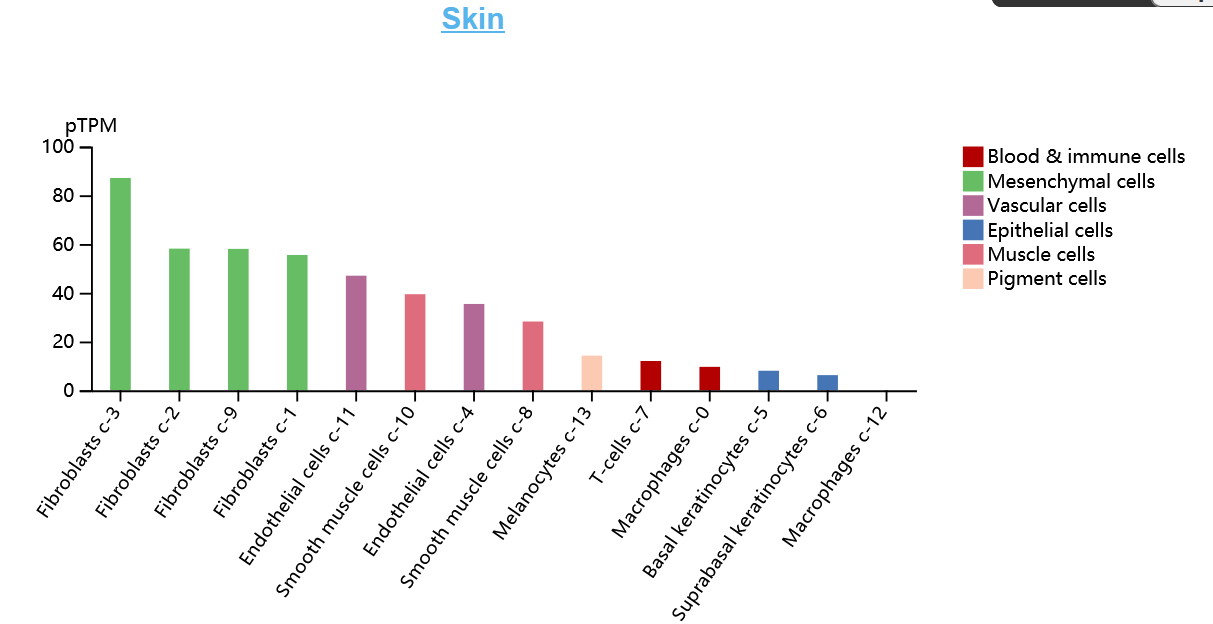

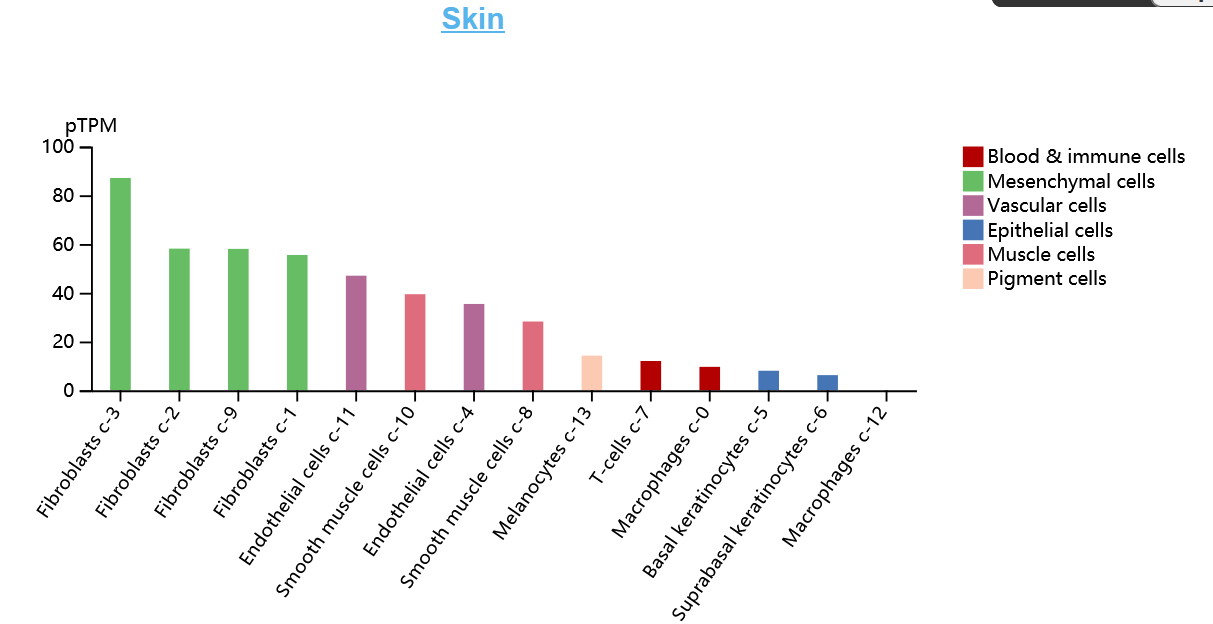


**Figure S2 Archived MXRA7 expression in various cells of different organs in Human Protein Atlas.** Each panel was a direct screenshot from the website without any modification, and the data were arranged according to pTPM from high to low in each cell cluster. Details of each dataset were available in the HPA webstie.


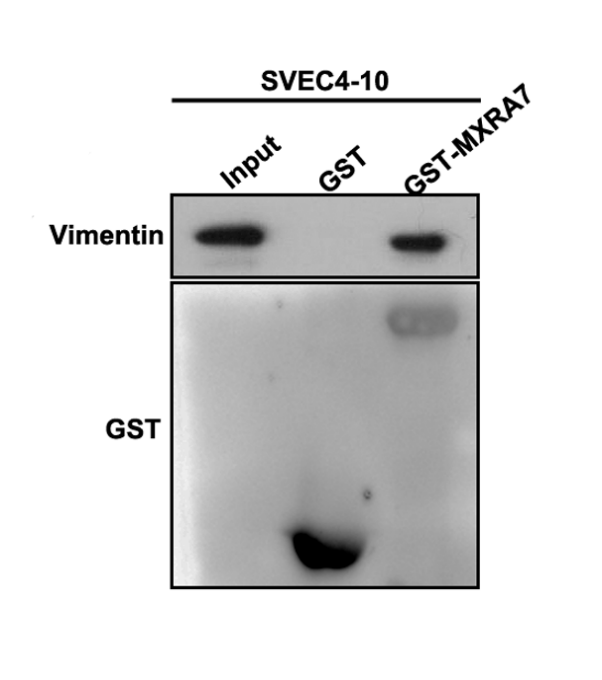


**Figure S3. GST pull-down analysis of the interaction between MXRA7 and Vimentin proteins by SDS-PAGE.** Vimentin is captured by GST-MXRA7 pull down from SVEC4-10 cell membrane lysate.


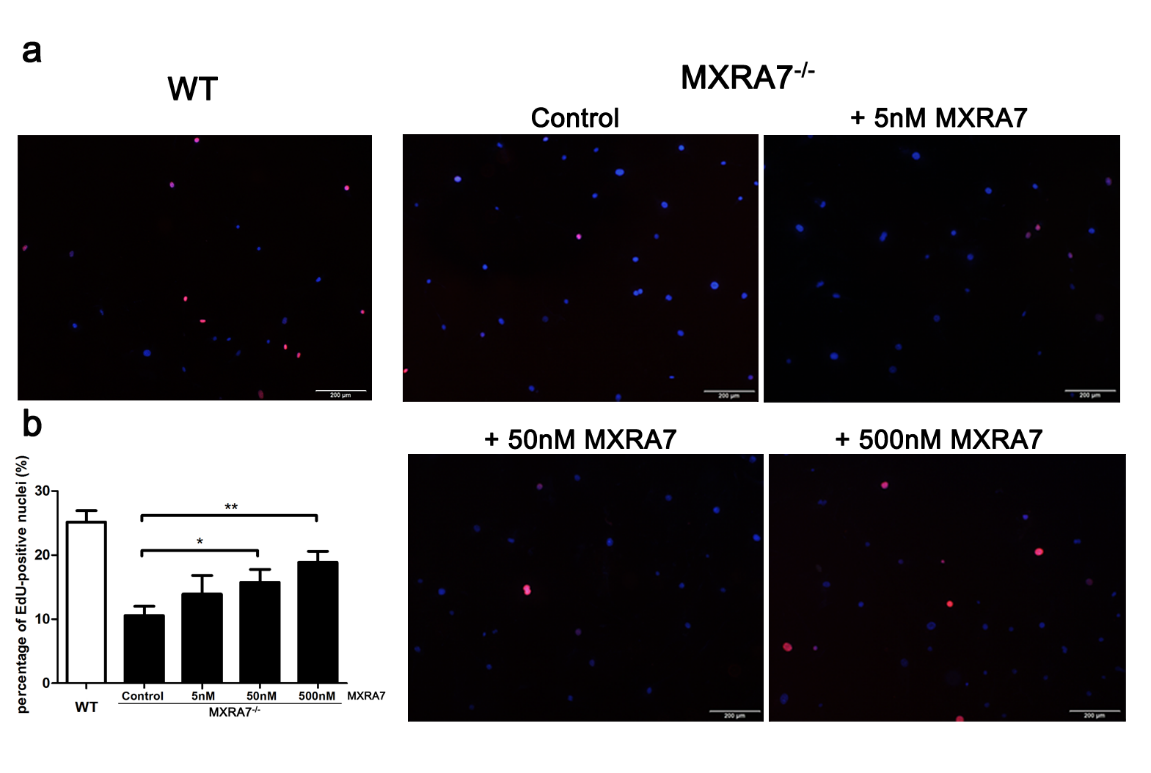


**Figure S4. EdU proliferation assay analysis of the effect of different concentrations of rmMXRA7 on the growth of MXRA7^-/-^** **fibroblast cells.** (a) Images of fibroblast cells labelled with EdU+ (red) and DAPI (blue). The EdU proliferation assay was performed 24 h after different concentrations of rmMXRA7 were added to the MXRA7^-/-^ fibroblast cells. (b) Percentage of cells labelled with EdU. *P<0.05; **P<0.01; ***P<0.001; (Student's t-test). The results are shown as mean±S.D.
